# *Escherichia coli* O157:H7 Acid Sensitivity Correlates with Flocculation Phenotype during Nutrient Limitation

**DOI:** 10.3389/fmicb.2017.01404

**Published:** 2017-07-26

**Authors:** Kathryn L. Kay, Frederick Breidt, Pina M. Fratamico, Gian M. Baranzoni, Gwang-Hee Kim, Amy M. Grunden, Deog-Hwan Oh

**Affiliations:** ^1^Department of Plant and Microbial Biology, North Carolina State University Raleigh, NC, United States; ^2^Food Science Research Unit, United States Department of Agriculture-Agriculture Research Service Raleigh, NC, United States; ^3^United States Department of Agriculture, Agricultural Research Service, Eastern Regional Research Center Wyndmoor, PA, United States; ^4^Department of Food Science and Biotechnology, College of Bioscience and Biotechnology, Kangwon National University Chuncheon, South Korea

**Keywords:** STEC, acid resistance, nutrient limitation, curli, GcvB

## Abstract

Shiga toxin producing *Escherichia coli* (STEC) strains vary in acid resistance; however, little is known about the underlying mechanisms that result in strain specific differences. Among 25 STEC O157:H7 strains tested, 7 strains flocculated when grown statically for 18 h in minimal salts medium at 37°C, while 18 strains did not. Interestingly, the flocculation phenotype (cells came out of suspension) was found to correlate with degree of acid sensitivity in an assay with 400 mM acetic acid solution at pH 3.3 targeting acidified foods. Strains exhibiting flocculation were more acid sensitive and were designated FAS, for flocculation acid sensitive, while the acid resistant strain designated PAR for planktonic acid resistant. Flocculation was not observed for any strains during growth in complex medium (Luria Bertani broth). STEC strains B201 and B241 were chosen as representative FAS (2.4 log reduction) and PAR (0.15 log reduction) strains, respectively, due to differences in acid resistance and flocculation phenotype. Results from electron microscopy showed evidence of fimbriae production in B201, whereas fimbriae were not observed in B241.Curli fimbriae production was identified through plating on Congo red differential medium, and all FAS strains showed curli fimbriae production. Surprisingly, 5 PAR strains also had evidence of curli production. Transcriptomic and targeted gene expression data for B201 and B241indicated that *csg* and *hde* (curli and acid induced chaperone genes, respectively) expression positively correlated with the phenotypic differences observed for these strains. These data suggest that FAS strains grown in minimal medium express curli, resulting in a flocculation phenotype. This may be regulated by GcvB, which positively regulates curli fimbriae production and represses acid chaperone proteins. RpoS and other regulatory mechanisms may impact curli fimbriae production, as well. These findings may help elucidate mechanisms underlying differences among STEC strains in relating acid resistance and biofilm formation.

## Introduction

Shiga toxin producing *Escherichia coli* (STEC) O157:H7 and some related serotypes are of particular interest in food safety and public health due to their ability to colonize a human host with a low infectious dose, as little as 2–2,000 cells for foodborne outbreak strains, and cause severe illness (Buchanan and Doyle, 1997). *E. coli* O157:H7 and other STEC produce Shiga toxins, the most critical virulence factor involved in inducing gastrointestinal illness characterized by hemorrhagic colitis, which may develop into hemolytic uremic syndrome (HUS) and can ultimately result in death (Buchanan and Doyle, 1997; Park et al., 2001; Pennington, 2010). Previous work has identified STEC strains as acid resistant pathogens of concern for acidified foods (Breidt et al., 2007, 2013). Acid and acidified food products are defined as foods having a pH of 4.6 or lower, which prevents residual spores from germinating as mandated in the United States Code of Federal Regulations (21 CFR 114; US Food Drug Administration, 2012). These foods contain a variety of organic acids, which contribute to flavor and have antimicrobial effects (Breidt et al., 2004; Oh et al., 2009).

Weak acids, such as acetic acid and lactic acid, are able to diffuse across the bacterial cell membranes and dissociate intracellularly because the intracellular pH is higher than the environmental pH. The acidification of the cell cytoplasm and intracellular accumulation of acid anions can lead to cell death, but cell death due to acid stress is also dependent on other factors such as, acid type, pH, temperature, and growth phase (Lin et al., 1996; Diez-Gonzalez and Russell, 1997, 1999; Castanie-Cornet et al., 1999; Breidt et al., 2004). Disease outbreaks of *E. coli* O157:H7 from acid and acidified foods, such as the multi-state outbreak in apple cider, have made the relationship between the antimicrobial effects of weak acids and *E. coli*'s acid resistance systems an important concern (Miller and Kaspar, 1994; Buchanan and Doyle, 1997; Rangel et al., 2005).

Acid habituation, induced by growing cells in mildly acidic medium, results in higher survival rates in response to acid challenge compared to cells grown in neutral pH media (Buchanan and Edelson, 1996; Brudzinski and Harrison, 1998). There are multiple acid resistance systems (designated AR1 through AR4) in *E. coli*. AR1 was observed in stationary cells grown in pH 5.5 buffered Luria broth (Bearson et al., 2009). These cells were able to survive acid challenge whereas cells grown at higher pH could not. The RpoS sigma factor and the F_1_F_0_ ATPase are required for this system (Lin et al., 1996). Other acid resistance mechanisms require decarboxylase and antiporter systems, based on glutamate (AR2) or arginine (AR3) substrates, to effectively export protons out of the cell and raise the intracellular pH (Lin et al., 1995; Hersh et al., 1996; Castanie-Cornet et al., 1999; Iyer et al., 2003; Bearson et al., 2009). The activity of these acid resistance systems correlates with internal pH and the optimal pH function of the decarboxylases (Foster, 2004). A lysine decarboxylase system (AR4) has also been identified, but it is not as effective as AR2 or AR3 in raising intracellular pH (Iyer et al., 2003).

RpoS is activated during stationary phase or environmental stresses, such as, acid shock, to induce stress response genes. In *Salmonella enterica* serovar Typhimurium, it was found that RpoS expression affects the cell's acid stress response for all AR systems (Lin et al., 1995). Active RpoS has been found to be essential for AR1 in *E. coli*, and RpoS mutants appear to indirectly affect AR2 and AR3 decarboxylase activity (Lin et al., 1996; Castanie-Cornet et al., 1999). RpoS also plays an important role in the regulation of biofilm formation, including the formation of extracellular curli amyloid protein fibers (Uhlich et al., 2013).

Curli expression by STEC is characterized by adhesive fimbrial aggregates that can result in biofilm production, contributing to the transition of motile cells to the sessile phenotype during environmental stress. Regulation of curli is complex, mediated by RpoS, regulatory small RNAs (sRNA), cross-talk with other surface fimbriae, and other regulatory molecules acting at both the transcription and translational levels (Beloin et al., 2008; Pesavento et al., 2008; Boehm and Vogel, 2012; Lloyd et al., 2012a). Carbon source quality, osmotic imbalance, and nutrient deprivation activate regulatory small RNAs (McaS, OmpR, and GcvB) that target curli synthesis at the translational level (Vidal et al., 1998; Chen et al., 2002; Jorgensen et al., 2012). Of particular interest, GcvB acts as a global repressor of amino acid metabolism, and transcription of *gcvB* is dependent on the concentration of environmental glycine. GcvB is also known to be a regulator of acid induced chaperone proteins (Urbanowski et al., 2000). If the concentration of environmental glycine is high, *gcvB* transcription may be upregulated, repressing expression of *csgD* and the curli structural genes *csgBA*, as well as inducing acid stress chaperone proteins encoded by *hdeAB*. GcvB has also been found to increase expression of RpoS under acid stress (Jin et al., 2009; Stauffer and Stauffer, 2012a).

A correlation between the production of curli fimbriae and acid resistance has been observed previously in *E. coli* O157:H7 strains, but the regulatory mechanisms responsible for coordinate regulation and the relationship to nutrient limitation are not understood. We proposed a hypothesis that nutrient limitation influenced expression of GcvB, RpoS, and other regulatory molecules that control expression of CsgD (curli) and HdeAB (acid chaperones). Because the differences in regulation of curli fimbriae were only observed for more acid sensitive cells grown in a minimal medium, gene expression of carbon storage, curli fimbriae, and changes in global regulators for acid resistance were targeted. These data will contribute to our understanding of how STEC strains respond differently to nutrient limitation and acid stress.

## Materials and methods

### Bacterial strains and culture conditions

All *E. coli* O157:H7 and K-12 strains used were obtained from the USDA Food Science Research Unit, Raleigh, NC culture collection and are listed in Table 1. Bacteria were grown statically in either 10 ml of Luria Bertani broth supplemented with 1% glucose (LBG; LB, Becton Dickinson, Sparks, MD) or a defined minimal medium (5X M9 Minimal Salts, Sigma-Aldrich, St. Louis, MO) supplemented with 1% glucose and 0.005% thiamine (M9GT) at 37°C for 18 h to induce acid resistance. Glycine (5 mM) was added to M9GT as indicated below. Cultures were harvested by centrifugation (5,000 × g, 10 min, 10°C, Sorvall Superspeed Centrifuge, SS-34 rotor, DuPont Instruments, Newton, CT), decanted, and re-suspended in 10 ml sterile saline [0.85% sodium chloride (NaCl)] twice prior to use.

**Table 1:** *Escherichia coli* O157:H7 isolates, acid resistance, and designated flocculation and curli phenotypes used in this study.

**Isolate**		**Source (reference)^a^**	**Reduction in log CFU/ml^b^**	**Phenotype^c^**	**Curli^d^**
**ID no**.	**Original ID no**.				
B055	USDA-ARS K-12	Human isolate	N/A	FAS	−
B201	SRCC 1675	Apple cider, October 2002	2.42 ± 0.05	FAS	++
B202	SRCC 1486	Salami, October 2002	0.29 ± 0.01	PAR	−
B204	SRCC 1941	Pork, September 2002	0.17 ± 0.01	PAR	+
B241	28RCI	Bovine carcass	0.15 ± 0.02	PAR	−
B244	3014-93	Human outbreak	0.15 ± 0.01	PAR	+
B245	3055-93	Human outbreak	0.26 ± 0.08	PAR	−
B246	3139-98	Human outbreak	1.06 ± 0.18	FAS	+
B247	3159-98	Human outbreak	0.26 ± 0.08	PAR	+
B249	3187-95	Human outbreak	0.17 ± 0.03	PAR	−
B250	3261-98	Human outbreak	0.17 ± 0.03	FAR	++
B251	3361-91	Human outbreak	0.16 ± 0.03	PAR	−
B263	RM1242	Human, sporadic, 1997	1.04 ± 0.09	FAS	+
B264	RM1484	Apple juice, associated with 1996outbreak	0.21 ± 0.03	PAR	+
B265	RM1918	Human, outbreak, 1999, lettuce	0.27 ± 0.03	PAR	−
B266	RM2189	Human, outbreak, 1999, taco meat	0.16 ± 0.05	FAR	+
B269	RM4263	Human, outbreak, 2000, waterborne	0.09 ± 0.02	PAR	+
B271	RM4406	Human, outbreak, 2003, leafy vegetable	1.00 ± 0.05	FAS	++
B273	RM4688	Human, outbreak, 2002, leafy vegetable	0.25 ± 0.01	PAR	−
B296	RM5279	Human, outbreak, 2005, leafy vegetable	0.36 ± 0.12	PAR	−
B301	RM5630	Water	0.11 ± 0.01	PAR	−
B306	RM5850	Water	0.19 ± 0.05	PAR	−
B307	RM5875	Water	1.03 ± 0.11	FAS	++
B309	RM5714	Water	0.49 ± 0.07	PAR	−
B311	RM6011	Human, outbreak, 2006, leafy vegetable	0.31 ± 0.03	PAR	−
B349	NMSLD-1	Spinach	0.28 ± 0.06	PAR	−

Identification of E. coli O157:H7 isolates;

a reference source (Oh et al., 2009);

b log reduction with standard error after acid challenge (400 mM acetic acid, pH 3.3, 30°C) after growth in LBG;

c acid sensitivity and flocculation phenotypes FAR (flocculating - acid resistant), PAR (planktonic - acid resistant) and FAS (flocculating - acid sensitive);

d *curli fimbriae production denoted by (−) indicating no curli production, (+) some curli expression, and (++) maximal expression of curli via colorimetric assay^d^*.

For acid challenge experiments, initial cell concentrations were approximately 10^9^ CFU/ml. After acid challenge (see below) cells were diluted 1:10 in 0.1 M 3 N-morpholino propane sulfonic acid buffer (MOPS; pH 7.2, Sigma Aldrich) to relieve acid stress, followed by serial dilution in sterile saline. Cells were enumerated on LB agar (Becton Dickinson) using a spiral plater (Autoplate 4000, Spiral Biotech, Inc., Norwood, MA) and an automated plate reader (QCount, Spiral Biotech), or by plating 10 μl onto 5 ml of LB agar in 24-well microtiter plate wells to estimate counted colony forming units (CFU).

For gene deletion experiments, bacterial strains were grown in LBG broth or on LB agar supplemented with antibiotics at the following concentrations, 50 μg/ml ampicillin and 25 μg/ml chloramphenicol. For gene deletion experiments, bacteria were grown aerobically in 1.0 ml of LBG at 37°C overnight at 1200 RPM in the Eppendorf Thermomixer R (Hamburg, Germany). Overnight cultures (30 μL) were re-inoculated in 1.0 ml fresh LBG and grown for 3 h at 37°C before electroporation.

### Curli and motility assays

Curli phenotype was determined for all strains of *E. coli* O157:H7 and a K-12 strain. Cultures were grown in LBG or M9GT broth at 37°C for 18 h, and then plated onto Congo red indicator (CRI) agar composed of 10 g/L casamino acids (Becton Dickinson), 1 g/L yeast extract (Becton Dickinson), and 20 g/L agar (Thermo Fisher Scientific, Fair Lawn, NJ) with 20 mg/L of Congo red (Polysciences, Inc., Warrington, PA) with and without 10 mg/L Coomassie Brilliant Blue G-250 (MP Biomedicals, Inc., Solon, OH; Uhlich et al., 2006). LB agar supplemented with 200 mg/L calcoflour white stain (Fluka Analytical), a UV fluorescent stain that binds to cellulose and chitin, plates were used to identify cellulose production (Hammar et al., 1995; Uhlich et al., 2006). Motility was determined using motility test medium (MTM; 3 g/L beef extract (Becton Dickinson), 10 g/L pancreatic digest of casein (Becton Dickinson), 5 g/L NaCl, and 4 g/L agar) stab cultures grown 18 h at 37°C (Atlas and Parks, 1993).

### Acid resistance assay

To determine acid resistance of STEC strains, acetic acid solutions were prepared as described previously (Oh et al., 2009). The pH of the glacial acetic acid solutions (400 mM) was adjusted to 3.3 with the addition of sodium hydroxide (1N). Cell suspensions were added (200 μl) into 1.8 ml of acetic acid solution in 12-well tissue culture plates. The 12-well tissue culture plate was incubated aerobically at 30°C for 25 min (Oh et al., 2009). After incubation, 20 μl of the cell suspension was transferred into 180 μl of 0.1 M MOPS followed by serial 10-fold dilutions and plating onto LB agar to determine CFU/ml as described above.

### Transmission electron microscopy

Transmission electron microscopy (TEM) was performed to confirm the presence or absence of flagella in acid resistant and sensitive strains. Cultures were grown statically in 10 ml of M9GT or LBG at 37°C for 18 h, harvested by centrifugation (5,000 × g, 10 min, 10°C), and then re-suspended in 1 ml sterile saline and serially diluted into 3% glutaraldehyde fixative at 4°C for 4 h. Cells were harvested in a microcentrifuge (10,000 × g, 10 min, 22°C, Spectrafuge 24D Digital Microcentrifuge, Labnet International, Inc., Edison, NJ), re-suspended in 0.1 M sodium acetate buffer at pH 7.0, and transported to North Carolina State University's Center for Electron Microscopy for processing. Cells were stained with 1 and 0.5% phosphotungstic acid (PTA; Ernest F. Fullan, Inc., Schenectady, NY) on 400-mesh carbon grids (Ladd Research Industries, Williston, VT). Cells on the grids were imaged at a magnification of either 12,000X or 15,000X on a JEOL JEM 1200 EX (JEOL U.S.A., Peabody, MA) transmission electron microscope at 80 kV.

### RNA extraction

Two volumes of RNA-protect Bacterial Reagent (Qiagen Sciences, Inc., Germantown, MD) were added to one volume (1.5 ml) of culture (approximately 10^9^ CFU/ml) grown statically in M9GT ± 1% glycine at 37°C for 18 h. Cells were incubated at room temperature for 5 min and harvested by centrifugation (5,000 × g, 10 min, 10°C). Total RNA was extracted using the RNeasy Mini Kit (Qiagen Sciences) with an additional on-column DNaseI digestion following the manufacturer's protocol. For transcriptomic analysis, a higher yield of total RNA was required, and thus the RNeasy Midi Kit (Qiagen Sciences) was used. DNA and ribosomal RNA (rRNA) were removed from the extracted RNA using the TURBO DNA-free kit (Thermo Fisher Scientific) and Ribo-Zero rRNA Removal Gram-Negative kit (Illumina, Inc., San Diego, CA). The rRNA-depleted RNA samples were concentrated and purified using Zymo RNA Clean & Concentrator columns (Zymo Research, Irvine, CA). Concentration and quality of RNA were determined using the RNA Nano-chip and the RNA Pico-chip on an Agilent 2100 Bioanalyzer (Agilent, Santa Clara, CA) for transcriptomic analysis, or a Nanodrop ND-1000 UV-visible wavelength spectrophotometer (NanoDrop Technologies, Wilmington, DE) for RT-qPCR experiments.

### RNA sequencing and transcriptomic analysis

One library for each strain (B201, B241, and B250) was prepared using the Ion Total RNA-Seq Kit v2 (Thermo Fisher Scientific) following the manufacturer's instructions with one exception: incubation time for RNA shearing was reduced to 2.5 min. The final cDNA library was quantified with a 2100 Bioanalyzer using a High Sensitivity DNA Chip (Agilent). Library enrichment was carried out with an Ion PGM™ Template OT2 200 kit and Ion OneTouch™ 2 system (Thermo Fisher Scientific). Each library was sequenced with an Ion 318 Chip using an Ion PGM 200 Sequencing kit (Thermo Fisher Scientific) on the Ion Personal Genome Machine (Thermo Fisher Scientific). RNA-Seq data were analyzed by ProteinCT Biotechnologies (Madison, WI, USA). Briefly, raw reads were mapped to the *E. coli* O157 B201 and B241 genome sequences (Baranzoni et al., 2016) using the Subjunc program from the Subread-1.4.6 package (http://bioinf.wehi.edu.au/subread). Raw gene counts were generated by FeatureCounts from the Subread package, and differential expression was calculated using the Limma package (https://bioconductor.org/packages/release/bioc/html/limma.html). Transcriptomics data are available under NCBI BioProject number PRJNA381969.

### Quantitative real-time reverse transcriptase-PCR (RT-qPCR)

For quantitative analysis of gene expression, the Bio-Rad One-Step RT-qPCR kit (Bio-Rad, Hercules, CA) was used to synthesize cDNA from cellular RNA (2 μl template) extracted from *E. coli* B201, B241, and B250. Primers for selected transcripts (Table 2) were designed with the IDT Real Time PCR Tool. Gene expression analysis was performed on the Bio-Rad CFX96 Touch Real-Time PCR Detection System (Bio-Rad). The Bio-Rad One-Step RT-qPCR kit (Bio-Rad) was tested without RNA, without primers, and without reverse transcriptase as negative controls to determine the efficacy of the kit, the presence of additional genomic DNA, and potential primer-dimer interactions, respectively. Target genes were normalized using the *tus* gene as an endogenous control (Crépin et al., 2012; Porcheron et al., 2014). Cycle threshold (C_T_) values and relative fold change were calculated as (2^−ΔΔ*C_T_*^), using an average of three independent replications (Livak and Schmittgen, 2001).

**Table 2:** The primer sequences for RT-qPCR and chloramphenicol linear cassette.

**Target gene**	**Primer code**	**Sequence**	**Amplicon length^a^**
*Tus*	tus_For1	5′-CACAGAACGCGAAGTTA-3′	115
	tus_Rev1	5′-GCAATCAGTGGTGTAGG-3′	
*csgA*	csgA_For1	5′-GCTGATGCTCGTAACTC-3′	152
	csgA_Rev1	5′-GAGTCTTTACCGTTCCAC-3′	
*csgB*	csgB_For1	5′-GCCAATGATGCCAGTAT-3′	156
	csgB_Rev1	5′-TTGTGTCACGCGAATAG-3′	
*csgD*	csgD_For1	5′-AGTAAGGAGGGCTGATT-3′	143
	csgD_Rev1	5′-CCATGGAGGATCAAGAAC-3′	
	csgDKO_ChlorF50	5′-CGAACAGAAATTCTGCCGCCACAATCCAGCGTAAATAACGTTTCATGGCTTGTGACGGAAGATCACTTCG-3′	1,111
	csgDKO_ChlorR50	5′-TGCTTCTATTTTAGAGGCAGCTGTCAGGTGTGCGATCAATAAAAAAAGCGACCAGCAATAGACATAAGCG-3′	
	csgDSeq_For1	5′-ACTTCTACCTCAACGGCGTG-3′	776, 1,136
	csgDSeq_Rev1	5′-GCTGTCAGGTGTGCGATCA-3′	
*csrA*	csrA_For1	5′-CAGCCTGGATACGCTGGTAG-3′	147
	csrA_Rev1	5′-CTCGTCGAGTTGGTGAGACC-3′	
*csrB*	csrB_For1	5′-CAGGATGGAGAATGAGAAC-3′	106
	csrB_Rev1	5′-CTATTGCTTCCTGCTCAC-3′	
*csrC*	csrC_For1	5′-GCGAAGACAGAGGATTG-3′	149
	csrC_Rev1	5′-CCTGACTCATAACCCTTAAC-3′	
*csrD*	csrD_For1	5′-CTGGTTCTCCGTTCGCTTCT-3′	206
	csrD_Rev1	5′-TTGAACTTGCAGAGGCCGAT-3′	
*cycA*	cycA_For1	5′-AACAGCGTCCTCATCTA-3′	121
	cycA_Rev1	5′-GTGTCATCTTCCAGTGTC-3′	
*flhC*	flhC_For1	5′-GTGCGGTTTGTTGAAAG-3′	121
	flhC_Rev1	5′-ATGGCGGTTGACATAAG-3′	
*flhD*	flhD_For1	5′-CCGCTATGTTTCGTCTC-3′	100
	flhD_Rev1	5′-ACCAGCTGATTGGTTTC-3′	
*fliC*	fliC_ForA	5′-CGCGGAGTTCACATTTA-3′	140
	fliC_RevA	5′-CTAACGTTGCCGACTATAC-3′	
*gcvA*	gcvA_For1	5′-GAACACACCGGCAATAA-3′	119
	gcvA_Rev1	5′-GATCGTCAGGAAGATAAGC-3′	
*gcvB*	gcvB_For1	5′-CCTGAGCCGGAACGAAA-3′	106
	gcvB_Rev1	5′-GTCTGAATCGCAGACCAA-3′	
*gcvR*	gcvR_For1	5′-CCGTCATGTCAGTAGTTG−3′	100
	gcvR_Rev1	5′-ATTCCATGAACCGGAAAG-3′	
*glyA*	glyA_For1	5′-CCAGGAACAGATGGTTATC-3′	136
	glyA_Rev1	5′-CTGAAAGAAGCGATGGAG-3′	
*hdeA*	hdeA_For1	5′-GTACAAGCCTGAACGATAG-3′	154
	hdeA_Rev1	5′-GGACCTGTGAAGATTTCC-3′	
*hdeB*	hdeB_For1	5′-ATTCCTGGCAGGTCATA-3′	117
	hdeB_Rev1	5′-TTTCATCTCTCCGTAAAGC-3′	
*hfq*	hfq_For1	5′-GGGCAAATCGAGTCTTT-3′	126
	hfq_Rev1	5′-GGCGTTGTTACTGTGAT-3′	
*mlrA*	mlrA_For1	5′-CGAACGTGGATCAAAGAG-3′	162
	mlrA_Rev1	5′-CAGACAAATGGCGATGTA-3′	
*pgaA*	pgaA_For2	5′-GGTCAGACTGTCGTTTATG-3′	100
	pgaA_Rev2	5′-AGTACGGTCTGGGTTATC-3′	
*pgaB*	pgaB_For2	5′-GTGGATGCCGGTATTAAG-3′	113
	pgaB_Rev2	5′-GAGAGAGACGGTGATATTG-3′	
*pgaC*	pgaC_For2	5′-CGTCTATTTCTGGGTCTATC-3′	141
	pgaC_Rev2	5′-GCGTGTATGGTTTCCTC-3′	
*pgaD*	pgaD_For2	5′-GGGCGCTGTACAATAAG-3′	104
	pgaD_Rev2	5′-GAGCTCATCAGGTATTGC-3′	
*rpoS*	rpoS_For1	5′-CGAATAGTACGGGTTTGG-3′	154
	rpoS_Rev1	5′-CGTTGCTGGACCTTATC-3′	

List of primers designed for RT-qPCR and amplification of the chloramphenicol linear cassette, with 50 nt homology up and downstream of target genes as well as defined cat cassette homology (indicated by underlined nts) to T-SACK, needed for gene deletion mutants. Corresponding amplicon length for RT-qPCR targets, linear cassette, and sequencing primers are listed;

a *where sequencing primers have two amplicon lengths derived from the wild-type and respective gene deletion mutant strains*.

### Generation of a functional chloramphenicol cassette

Primers for gene deletion mutants (Table 2) were designed to produce a functional chloramphenicol linear cassette (1,011 bp). Linear cassette primers were designed with 50 bp homology directly up and downstream of the target insertion site and with flanking homology arms to a *cat* (CmR) region in T-SACK (Li et al., 2013). T-SACK, an *E. coli* strain carrying amplifiable drug resistance cassettes [*tetA-sacB, amp, cat*, and *kan*], was generously provided to us by Dr. Donald Court at the National Cancer Institute (Frederick, MD). Additional primers for gene sequencing were designed with the IDT Real Time PCR Tool (Table 2). Platinum PCR Supermix High Fidelity (Invitrogen, Carlsbad, CA) was used for amplification of the functional chloramphenical cassette. T-SACK DNA template was used for the initial amplification of the chloramphenicol cassette, but secondary amplification was performed with PCR product template for increased DNA concentrations (approximately 400 ng/μl). PCR products were purified to eliminate unincorporated primers, primer-dimers, and non-specific amplification products with the QIAquick PCR Purification Kit (Qiagen Sciences, Inc., Germantown, MD). DNA concentration and quality was determined using the Nanodrop ND-100 UV-visible wavelength spectrophotometer (NanoDrop Technologies, Wilmington, DE). Lastly, the chloramphenicol cassette was visualized by confirming the correct band size, 1,111 bp, through gel electrophoresis.

### Gene deletion mutants

The Quick & Easy *E. coli* Gene Deletion Kit (Gene Bridges, Heidelberg, Germany) was used according to manufacturer's protocol, with some exceptions, to produce gene deletion mutants. *E. coli* strains B201 and B241 were grown as stated for gene deletion experiments and transformed with 5 μl Red/ET expression plasmid carrying a functional *amp* region (pRedET, 20 ng/μl) by electroporation (Eppendorf 2510 Electroporator) at 1350 V, 10μF, 600 Ohms with a 5 ms pulse rate. Transformants (B201+pRedET and B241+pRedET) were selected on LB-ampicillin agar grown at 30°C overnight. Transformant colonies were grown as stated for gene deletion experiments, but at 30°C in LBG supplemented with ampicillin. Overnight cultures (30 μl) were re-inoculated in fresh media conditioned with ampicillin at 30°C for 2 h before inducing pRedET with 50 μl 10% L-arabinose and incubating at 37°C for 1 h. Induced cells were harvested in a cooled benchtop microcentrifuge (13,000 × g, 1 min, 4°C, Spectrafuge 24D Digital Microcentrifuge, Labnet International, Inc., Edison, NJ), decanted, and re-suspended in sterile ice cold de-ionized water three times before electroporation with the chloramphenicol cassette (1 μg/μl). Electroporated transformants were added to 1.0 ml LBG without antibiotics and incubated at 37°C for 3 h for recombineering to occur. Recombinant colonies were grown on LB agar conditioned with chloramphenicol at 37°C overnight.

### Gel electrophoresis and sequencing of gene deletion mutants

Gene deletion mutants were confirmed through colony PCR. Briefly, recombinant and wild-type colonies were re-suspended in 30 μl of de-ionized water and incubated at 98°C for 5 min to lyse cells. PCR was performed with Platinum PCR Supermix High Fidelity recombinant and wild-type DNA templates (2 μl), linear cassette primers, and sequencing primers. PCR products were visualized by agarose gel electrophoresis, ran at 65V for 100 min, and confirmed by band size by comparison with standards (accession number CP015020 and CP015023 for B201 and B241, respectively). Additionally, gene deletion mutants and wild-type target genes were confirmed through PCR product sequencing by Eton Biosciences, Inc. (Durham, NC). PCR product chromatographs were analyzed through FinchTV software (Perkin Elmer, Waltham, MA) and the corresponding sequences were aligned with B201 (accession number CP015020) and B241 (accession number CP015023) sequences through GenBank Blastn software (National Center for Biotechnology Information, Bethesda, MD; Baranzoni et al., 2016).

### Statistical analysis

All experiments were done with three or more independent replications. Statistical analysis for acid challenge and *C*_*T*_ data was performed in SAS (version 9.4 software, SAS Institute, Inc., Cary, NC). Differences (*p* < 0.05) in acid resistance between bacterial strains were determined using the student *t*-test. Distribution and variance was determined (*p* < 0.05) through the Shapiro-wilk and Fligner-Killeen tests, respectively. Differences (*p* < 0.05) in acid resistance between the presence or absence of flocculation or curli fimbriae production were determined using the Welch *t*-test, due to the data not being distributed normally and having heterogeneous variance. Differences (*p* < 0.05) between strain gene expression were analyzed with both Δ*C*_*T*_ and linearized ($2^{-{\Delta C}_{T}}$) values by student *t*-test controlling the False Discovery Rate using Benjamini-Hochberg correction.

## Results

### Characterization of flocculating and planktonic STEC strains

When grown in M9GT, the flocculation phenotype was found to correlate to acid sensitivity (*p* < 0.05). Planktonic strains were in general more acid resistant than flocculating strains, typically having less than a 1 log reduction in cell number in the acid resistance assay (Table 1). These strains were designated PAR (planktonic acid resistant). Flocculating strains had greater acid sensitivity, and were designated FAS (flocculating acid sensitive). Of the 26 *E. coli* strains tested 6 were defined as FAS, including a K-12 strain, while 18 were classified as PAR (Table 1). Two remaining strains (B250 and B266) had an intermediate phenotype and were designated FAR (flocculating acid resistant). B241 and B201 were chosen as representative PAR and FAS strains because of contrasting acid resistance, with log reduction values of 0.15 and 2.42, respectively, in the acetic acid assay (Table 1). B201 was isolated from apple cider, and B241 was a bovine isolate. These two O157:H7 strains have been sequenced (Baranzoni et al., 2016) and both STEC strains had similar genome size and content, and each strain harbored a large 81 kb (B241) or 95 kb (B201) plasmid.

**Figure 1:**
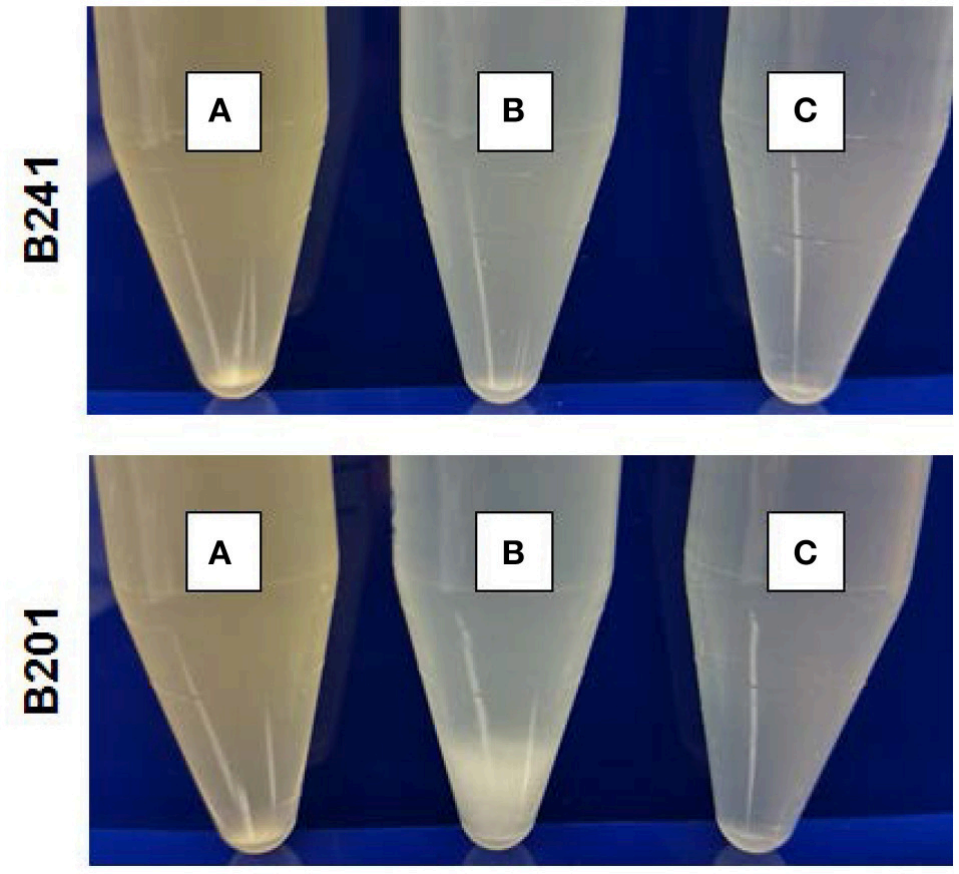
Growth of *E. coli* O157:H7 PAR strain B241 **(top)** and FAS strain B201 **(bottom)** in (A) LBG, (B) M9GT, and (C) M9GT supplemented with 1% glycine. Representative flocculation phenotype is displayed by FAS strain B201 in M9GT whereas flocculation is repressed with glycine supplementation.

Figure 1 shows the flocculation phenotype for representative strains B201 (FAS, 2.42 log reduction) and suspended phenotype for B241 (PAR, 0.15 log reduction) grown in M9GT and LBG. Additionally, acid resistance of 3 of the 26 strains B201, B241, and B250 (FAR, 0.17 log reduction) was tested when the strains were grown in M9GT with and without supplemental glycine. No significant difference was seen in viability between strains grown in M9GT and LBG. Flocculation was not apparent regardless of acid resistance phenotype in complex medium or M9GT with glycine supplementation (Figure 1).

### Curli expression for acid sensitive and resistant STEC

**Figure 2:**
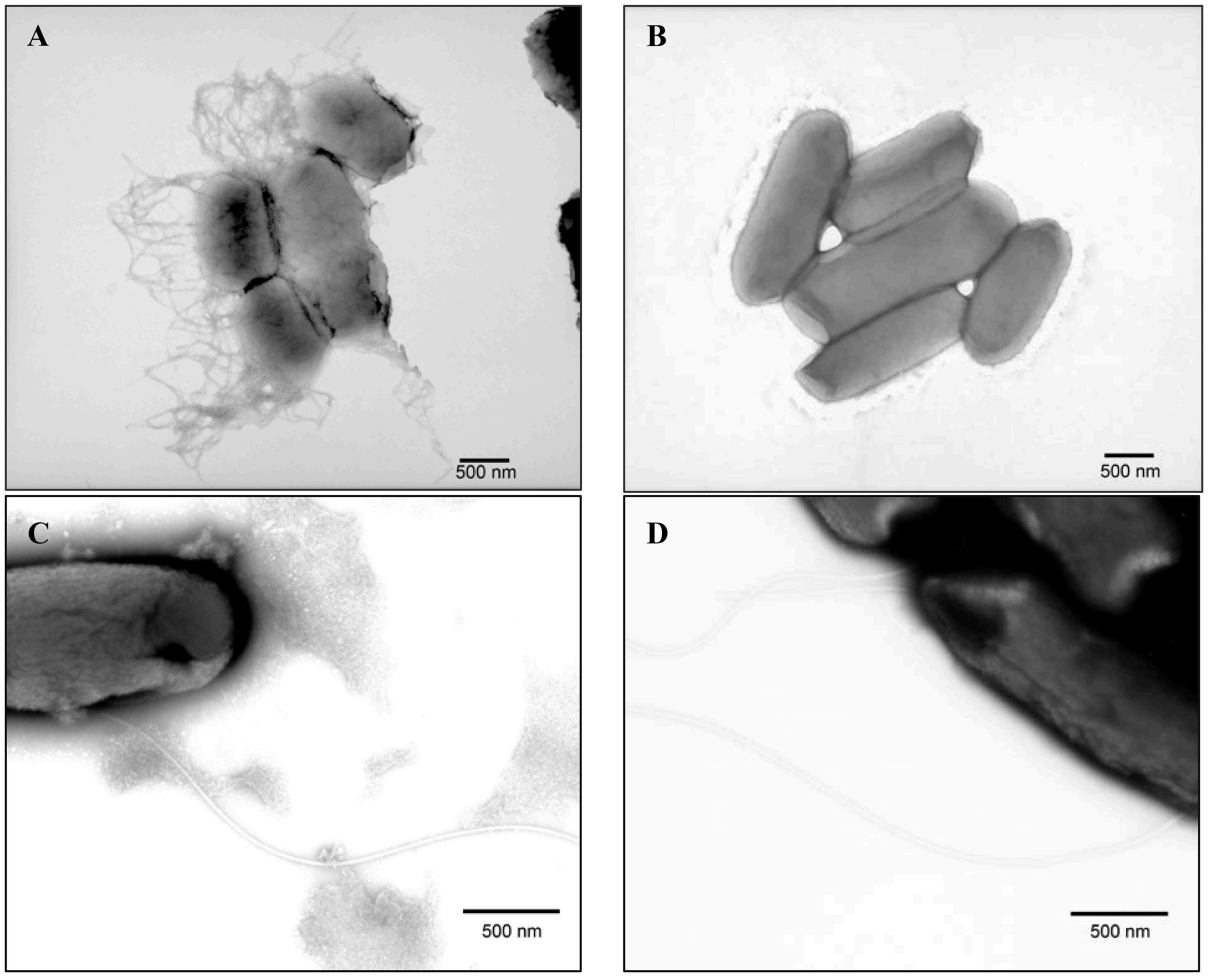
Transmission electron microscopy images of PTA stained **(A)** acid sensitive (B201) grown in M9GT exhibiting curli, **(B)** acid resistant (B241) grown in M9GT without curli, **(C)** acid sensitive (B201) grown in LBG exhibiting flagella, and **(D)** acid resistant (B241) grown in LBG exhibiting flagella.

Strains B201 and B241 were grown in both M9GT and LBG and subjected to TEM. We found that B201 cells grown in M9GT appeared in clumps with a network of fimbriae, and flagella were not observed (Figure 2A). These data indicated that B201 should be non-motile when grown in M9GT. Data from a motility test were inconclusive; however, B241 cells grown under the same conditions were not observed to have fimbriae or flagella (Figure 2B). However, B201 and B241 cells grown in LBG displayed flagella with no apparent fimbriae (Figures 2C,D, respectively). All strains were screened for the curli phenotype using CRI agar assay (Table 1). FAS strains all produced curli, and the curli phenotype was statistically associated (*p* < 0.05) with lower levels of acid resistance when compared to non-curli producing strains. Of the 20 acid resistant strains, 13 strains did not produce curli. Notably, acid resistant strain B250 exhibited high levels of curli production when compared to other resistant strains (Table 1). Interestingly, the calcofluor assay indicated that cellulose, which may be present during biofilm formation and associated with curli production, was not being produced by any of the strains (data not shown). Constructed curli deletion mutant B201Δ*csgD*, which did not express the curli regulatory protein CsgD, did not flocculate when grown in M9GT. Unsurprisingly, no difference in phenotype was observed for B241Δ*csgD* when grown in M9GT. Additional studies with B201Δ*csgD* and B241Δ*csgD* will be the subject to future work. These data support the hypothesis that curli fimbriae production was responsible for the flocculation seen in FAS STEC strains.

### The carbon storage, flagella, and chaperone proteins

To determine if carbon storage gene regulation correlated with curli fimbriae and flagella expression, we examined the expression of target genes for carbon storage regulation *csrABCD* (Dubey et al., 2003; Suzuki et al., 2006; Jonas and Melefors, 2009), flagella, and biofilm production *flhDC* (Wei et al., 2001; Yakhnin et al., 2013), *fliC* (Ogasawara et al., 2011), *mlrA* (Uhlich et al., 2013), and *pgaABCD* (Wang et al., 2005) in B201 and B241 grown in M9GT using a transcriptomic approach. We found that *csrB* and *csrC* (and other genes encoding sRNAs, including the regulatory sRNA *gcvB*, see below) were not present in the Limma package output, and quantitative expression studies for these genes were done using RT-qPCR. Expression of *csrA, flhC, flhD, mlrA*, and *pgaAB* was significantly different (*p* < 0.05) between B201 and B241, where *csrA* was down-regulated (−5.43 fold change) for B201 in comparison to B241 when grown in minimal medium and *flhC, flhD, mlrA*, and *pgaAB* were up-regulated (+1.76, +1.56, +1.27, +2.53, and +1.67, respectively). Fold-change values were determined for carbon storage, *csrBC*, and biofilm, *pgaCD*, genes (−1.33, −1.41, +1.85 and +1.76, respectively) although no statistical significance (*p* > 0.05) between mRNA levels was seen. Average *C*_*T*_ values and associated fold change are listed on Table 3. Transcriptomic analysis showed that some flagella genes, including the flagellar assembly protein FliH and the flagellar transcription regulatory protein FlhD were upregulated in B241 compared to B201, along with DnaK, ClpB, HtpG, and FimC precursor (Supplementary Table 1).

**Table 3:** Transcriptomics data for selected genes from strains B201 and B241.

**Gene**	**B201 (2^−Δ*C_T_*^)**			**B241 (2^−Δ*C_T_*^)**			**Fold change**	***p*-values (2^−Δ*C_T_*^)**	***p*-values (Δ*C_T_*)**
	**Mean**	***SD***	**CV (%)**	**Mean**	***SD***	**CV (%)**			
*csgA*	194.02	84.02	43	17.31	2.03	12	11.21	0.0479^*^	0.008^*^
*csgB*	74.56	58.06	78	11.10	1.70	15	6.71	0.2102	0.184
*csgD*	33.91	14.95	44	7.99	1.76	22	4.25	0.0822	0.025^*^
*csrA*	42.11	16.95	40	228.67	79.32	35	−5.43	0.0417^*^	0.025^*^
*csrB*	93,040.36	73,676.54	79	123,916.42	93,430.23	75	−1.33	0.7058	0.735
*csrC*	34.94	45.76	131	49.36	74.84	152	−1.41	0.7893	0.961
*csrD*	19.20	3.80	20	14.96	2.53	17	1.28	0.2449	0.241
*cycA*	25.32	19.33	76	41.45	22.31	54	−1.64	0.5049	0.438
*flhC*	13.24	0.90	7	8.48	0.59	7	1.56	0.0196^*^	0.011^*^
*flhD*	16.06	1.95	12	9.12	0.54	6	1.76	0.0225^*^	0.011^*^
*fliC*	18.82	1.77	9	14.88	3.68	25	1.26	0.2449	0.241
*gcvA*	13.20	6.55	50	17.74	8.46	48	−1.34	0.5929	0.642
*gcvB*	3.33	1.34	40	8.19	3.95	48	−2.46	0.1955	0.115
*gcvR*	53.51	40.37	75	37.90	5.91	16	1.41	0.5929	0.735
*glyA*	12.67	9.91	78	76.65	13.48	18	−6.05	0.0215^*^	0.032^*^
*hdeA*	17.50	10.54	59	979.68	209.54	21	−55.02	0.0196^*^	0.006^*^
*hdeB*	13.15	8.84	67	52.00	12.80	25	−3.95	0.0373^*^	0.040^*^
*hfq*	72.72	37.24	51	317.29	256.31	81	−4.36	0.2449	0.105
*mlrA*	13.56	0.68	5	10.70	0.74	7	1.27	0.0370^*^	0.025^*^
*pgaA*	26.03	6.09	23	10.27	0.18	2	2.53	0.0373^*^	0.011^*^
*pgaB*	14.61	2.45	17	8.74	0.83	9	1.67	0.0417^*^	0.029^*^
*pgaC*	47.74	51.68	108	25.85	22.18	86	1.85	0.5929	0.642
*pgaD*	16.41	4.55	28	9.30	2.38	26	1.76	0.1369	0.098
*rpoS*	54.68	25.33	46	162.95	30.76	19	−2.98	0.0370^*^	0.036^*^

*Data include: mean $2^{-{\Delta C}_{T}}$, standard deviation (SD), and coefficient of variation (CV). The fold-change values were normalization by tus gene expression using the ΔΔC_T_ method for comparison of B201 vs. B241. The bold values and asterisks denote statistically significant differences (p < 0.05) between strains for both $2^{-{\Delta C}_{T}}$ and ΔC_T_-values*.

### The curli regulatory network

To determine whether curli gene expression correlated with *gcvB* expression, we examined target genes *csgBA, csgD, gcvAB, gcvR* (Urbanowski et al., 2000)*, glyA* (Ghaz-Jahanian et al., 2013), and *hfq* (Mika and Hengge, 2013) in B201 and B241. In addition to curli expression, we examined targets regulated by GcvB, such as, acid stress response (*hdeAB* and *rpoS*) and amino acid transport (*cycA*) genes. Expression of *csgA, glyA, hdeAB*, and *rpoS* was significantly different (*p* < 0.05) between B201 and B241, as measured by RT-qPCR by the $2^{-{\Delta C}_{T}}$ transformation (Table 3). However, *csgD* was statistically significant (*p* < 0.05) when using the Δ*C*_*T*_ method as well. The results supported the conclusions from the CRI assays that the genes required for the curli phenotype, *csgA* and *csgD*, were up-regulated (+11.21 and +4.25 fold change, respectively) for B201 in comparison to B241. Transcription levels for *glyA* (involved in glycine production and transport) were down-regulated (−6.05 fold change) in B201. Expression of *rpoS* was also down-regulated (−2.98 fold change) for B201 in comparison to B241. Furthermore, expression of *hdeAB* was down-regulated (−55.02 and −3.95 fold change, respectively). Fold-change values were determined for *csgB, gcvAB, gcvR*, and *hfq* genes (+6.71, −1.34, −2.46, +1.41 and −4.36 fold change, respectively), yet statistical analysis indicated that there was no significance (*p* > 0.05) between treatments. RNA-Seq transcriptomic data showed that *csgBA* were up-regulated, whereas *hdeAB* were down-regulated for B201 in comparison to B241. In addition, the glutamate decarboxylase (AR2) genes were upregulated in B241 (Supplementary Tables 1, 2). As expected, the genes showing the largest decrease in expression in B241 compared to B201 included *csgA* and *csgB* (Supplementary Table 2).

### Effect of glycine on curli expression

The glycine cleavage system, dependent on glycine concentrations in the environment, is known to regulate both curli and acid chaperone protein expression (Urbanowski et al., 2000; Jin et al., 2009; Stauffer and Stauffer, 2012a), thus addition of glycine to FAS strains was performed to determine if curli production persisted. The curli phenotype for B201 was not observed when glycine was added to M9GT medium (Figure 1), and similar results were observed for FAR strain B250 which had intermediate acid resistance-flocculation phenotype (Table 1). As expected, the addition of glycine to M9GT cultures of B241 had no effect (Figure 1). We examined the effects of exogenous glycine on the glycine cleavage system genes, curli expression, and acid induced chaperone proteins (Table 4). Expression of *csgA, csgD, gcvA, mlrA*, and *rpoS* was up-regulated in B201 compared to B201 supplemented with glycine (+1.07, +1.41, +1.66, +2.12, and +3.29 fold change, respectively). As expected, expression of *cycA, gcvB, gcvR, glyA, hdeAB, and hfq* was down-regulated (−1.32, −9.66, −1.11, −1.37, −12.65, −1.10, and −2.07 fold-change, respectively) in B201. Unexpectedly, *csgB* was also down-regulated (−1.39 fold-change). However, acid challenge of B201 in M9GT showed similar log reduction values with and without glycine supplementation (1.52 log reduction in M9GT and 1.45 log reduction in M9GT supplemented with glycine, data not shown).

**Table 4:** Transcriptomics data for selected genes from strain B201 grown in M9GT and M9GT + glycine.

**Gene**	**B201 M9GT (2^−Δ*C_T_*^)**			**B201 M9GT + glycine (2^−Δ*C_T_*^)**			**Fold-change**	***p*-value (2^−Δ*C_T_*^)**	***p*-value (Δ*C_T_*)**
	**Mean**	***SD***	**CV (%)**	**Mean**	***SD***	**CV (%)**			
*csgA*	217.77	28.53	13	204.24	256.18	125	1.07	0.932	0.640
*csgB*	74.26	51.13	69	103.17	135.82	132	−1.39	0.892	0.983
*csgD*	37.82	4.19	11	26.91	15.03	56	1.41	0.578	0.615
*cycA*	26.45	11.30	43	34.79	13.07	38	−1.32	0.651	0.640
*gcvA*	14.60	5.43	37	8.81	4.69	53	1.66	0.578	0.614
*gcvB*	3.78	1.03	27	36.48	22.28	61	−9.66	0.416	0.117
*gcvR*	54.45	13.66	25	60.22	30.35	50	−1.11	0.892	0.983
*glyA*	12.45	3.26	26	17.05	6.12	36	−1.37	0.578	0.640
*hdeA*	19.18	3.65	19	242.60	371.09	153	−12.65	0.578	0.615
*hdeB*	11.27	4.25	38	12.44	7.34	59	−1.10	0.892	0.983
*hfq*	78.93	11.17	14	163.23	121.93	75	−2.07	0.578	0.640
*mlrA*	17.22	7.35	43	8.12	1.91	24	2.12	0.464	0.374
*rpoS*	62.93	23.74	38	19.11	8.62	45	3.29	0.416	0.129

*Data include mean $2^{-{\Delta C}_{T}}$, standard deviation (SD), and coefficient of variation (CV). The fold-change values were normalized by tus gene expression using the ΔΔC_T_ method for comparison of B201 in M9GT vs. M9GT+glycine*.

### Regulation of curli expression in strain B250

We measured expression of selected curli and chaperone target genes in strain B250, which was determined to be acid resistant, but exhibited the flocculation phenotype (Table 1). RT-qPCR data revealed that expression of *csgA, cycA, gcvR, glyA, hdeAB, hfq*, and *rpoS* was significantly different (*p* < 0.05) from B241, where *csgA* exhibited a +3.69 fold change in B250 in comparison to B241, yet no significant difference (*p* > 0.05) was seen in *csgB* or *csgD* expression (+9.15 and −1.07 fold change, respectively; Table 5). Expression of *cycA* and *glyA* was down-regulated (−3.50 and −6.75 fold change, respectively) in B250 when compared to B241, which was consistent with the data for strains B201 and B241. Additionally, *gcvR* and *hfq* expression was down-regulated (−1.86 and −14.77 fold change, respectively) in B250, although no statistically significant difference (*p* > 0.05) was seen in *csgD, gcvA*, or *gcvB* expression between treatments. Expression of *hdeAB* and *rpoS* was down-regulated (−87.80, −5.11, −12.55 fold change, respectively) in B250 in comparison to B241 which may account for the differences in acid sensitivity.

**Table 5:** Transcriptomics data for selected genes from strains B241 and B250.

**Gene**	**B250 (2^−Δ*C_T_*^)**			**B241 (2^−Δ*C_T_*^)**			**Fold-change**	***p*-values (2^−Δ*C_T_*^)**	***p*-values (Δ*C*_T_)**
	**Mean**	***SD***	**CV (%)**	**Mean**	***SD***	**CV (%)**			
*csgA*	53.49	23.93	45	14.48	0.78	5	3.69	0.104	0.015^*^
*csgB*	85.93	108.23	126	9.39	1.90	20	9.15	0.340	0.120
*csgD*	6.32	0.86	14	6.79	1.91	28	−1.07	0.780	0.874
*cycA*	9.94	2.51	25	34.79	19.43	56	−3.50	0.173	0.046^*^
*gcvA*	7.19	0.55	8	15.16	7.67	51	−2.11	0.190	0.161
*gcvB*	2.87	0.38	13	7.00	3.77	54	−2.44	0.190	0.079
*gcvR*	17.04	3.21	19	31.77	4.55	14	−1.86	0.026^*^	0.017^*^
*glyA*	9.50	2.16	23	64.15	8.95	14	−6.75	0.003^*^	0.002^*^
*hdeA*	9.31	1.93	21	817.16	123.86	15	−87.80	0.003^*^	<0.0001^*^
*hdeB*	8.55	2.21	26	43.69	11.16	26	−5.11	0.019^*^	0.005^*^
*hfq*	18.26	4.91	27	269.78	222.82	83	−14.77	0.190	0.015^*^
*mlrA*	8.97	1.55	17	9.02	1.03	11	−1.01	0.977	0.933
*rpoS*	11.01	3.10	28	138.10	33.29	24	−12.55	0.012^*^	0.002^*^

*Data include mean $2^{-{\Delta C}_{T}}$, standard deviation (SD), and coefficient of variation (CV). The fold-change was calculated with normalization expression data by tus gene expression using the ΔΔC_T_ method for comparison of B241 vs. B250. The bold values and asterisks denote statistically significant differences (p < 0.05) between strains for both $2^{-{\Delta C}_{T}}$-and ΔC_T_-values*.

## Discussion

All strains grew normally in LBG (a complex medium), however flocculation and curli production was found to correlate with acid sensitivity in a minimal medium, M9GT. However, two of the more acid resistant strains, B250 and B266, flocculated when grown in M9GT (minimal medium). Our findings agree with previous research that determined that *E. coli* does not express curli in static broth (LBG) or agar plates at 37°C (Barnhart and Chapman, 2006). The flocculation phenotype described in our work has been seen previously under environmental stress, including LB supplemented with NaCl, for the Shiga-toxin producing *E. coli* O111 serogroup (Diodati et al., 2015). In that study, environmental and outbreak strains displayed the aggregative curli phenotype, which was proposed to be related to biofilm formation, while sporadic-case strains did not. Furthermore, our curli gene deletion mutant, B201Δ*csgD*, was unable to express curli and did not flocculate when grown in M9GT. This observation proves that curli production is responsible for the flocculation phenotype observed in acid sensitive *E. coli* O157:H7 strains. In a study with *E. coli* O157:H7, curli production by strains grown in LB supplemented with NaCl was found to correlate with acid resistance (Carter et al., 2011). The data showed a 5-fold increase in the survival rate of non-curli variants after 6 h incubation in acidified broth when compared to curli variants challenged under the same conditions, although no regulatory link was proposed for curli and acid resistance. Carter et al. (2011) suggested that non-curli variants may be better fit for host colonization due to its acid resistance properties, whereas curliated *E. coli* O157:H7 may mediate attachment better to environmental surfaces such as, soils and plant tissues. Curli production may compensate for the absence of other fimbriae, such as, long polar fimbriae (Lpf), involved in colonization of human intestinal epithelial cells. For example, enhanced curli production and adhesion to cultured intestinal epithelial cells was seen in *E. coli* O157:H7 *lpfA1* and *lpfA2* deletion mutants after exposure to T84 cells (Lloyd et al., 2012a,b). Although the absence of Lpf did not activate curli production itself, alternative structures may be required under various environmental pressures. This transition suggests a regulatory connection between curli and Lpf, but specific mechanisms are currently unknown (Lloyd et al., 2012a,b). Using a proteomic approach (Gunther et al., 2014), Gunther and colleagues proposed a regulatory connection between the *gad* genes and the curli phenotype. Our transcriptomic data showed that glutamate decarboxylase genes were among those showing the largest increase in expression (Supplementary Table 1) for strain B241 compared to strain B201, which could contribute to the PAR phenotype we observed in M9GT, although further research would be needed to confirm this observation.

The carbon storage network of *E. coli* has been found to promote glycolysis and flagella production during growth in a complex medium, but gluconeogenesis and biofilm formation were stimulated by low pH and acetic acid (Suzuki et al., 2002; Mondragón et al., 2006; Jonas and Melefors, 2009; Chavez et al., 2010). Due to the glucose consumption during growth in M9GT, organic acid (primarily D-lactic acid) is produced by *E. coli*, which lowers the pH. These conditions led us to investigate *csr* genes in relation to the flocculation phenotype. We hypothesized that there would be increased expression of the sRNAs (CsrB and CsrC) that would affect other *csr* genes in FAS strain B201 compared to PAR strain B241. We found that the relative expression of *csrA, flhDC, fliC*, and *pgaAB* (for flagella and biofilm formation), was significantly different between FAS and PAR strains. Expression of *csrBC*, sRNA regulators, was not significantly different between B201 and B241 (Table 3). These data agree with other studies showing that decreased *csrA* expression correlated with increased biofilm through poly-N-acetylglucosamine production. Interestingly, we report decreased *csrA* expression with increased expression of flagella genes, *flhDC*. This appears to contradict reported findings from other studies on flagella formation regulation (Wei et al., 2001; Wang et al., 2005). We found that the carbon storage network affects biofilm formation and probably contributes to the flocculation phenotype, although we did not see regulation through *csrC* and *csrB*, small changes in sRNAs may account for large changes in target gene expression. It is likely that other changes in carbon metabolism are affecting this system that we did not evaluate.

For quantitative PCR data, the cycle threshold, or Δ*C*_*T*_, value is an exponential number based on a log-linear plot of the PCR signal vs. the cycle number. While some authors suggest statistical analysis on normalized untransformed Δ*C*_*T*_-values (Yuan et al., 2006), Livak and Schmittgen (2001) suggest performing statistical analysis with normalized exponential values, because the untransformed Δ*C*_*T*_-values may introduce more sample-to-sample variation. We found that the statistical significance for fold change for gene expression varied depending on method of calculation (transformed vs. untransformed), including *csgD* for comparisons between B201 and B241, as well as *csgA, csgD*, and *hfq* in comparison between B241 and B250. Changes in gene expression for these genes were statistically significant when analyzed with Δ*C*_*T*_-values but were not when analyzed with the $2^{-{\Delta C}_{T}}$-values. Because *csgD* appears to be a central regulatory molecule in acid resistance and curli production (influencing g*adAB* and *csgBA* expression, respectively), we therefore included both analyses in our data (Tables 3–5). Further work may be necessary to determine the significance of these observations, perhaps with increased replication of measurements.

Curli production by *E. coli* may aid biofilm formation under various environmental stresses (Olsén et al., 1989; Vidal et al., 1998; Uhlich et al., 2006; Ogasawara et al., 2007). The results of the CRI assay provide evidence for curli production by acid sensitive strains (Table 1). Although the K-12 strain we tested was considered acid sensitive, the CRI assay did not provide evidence for curli production. This is likely because previous research has found that *E. coli* K-12 produces curli at 37°C, but not on agar plates at this temperature (Barnhart and Chapman, 2006). Interestingly, *mlrA* expression, a MerR-like receptor controlled by RpoS that activates CsgD expression (Chen et al., 2013; Uhlich et al., 2013), was only significantly different between B201 and B241 despite the differences seen in *rpoS* expression between B241 and B250 (Tables 3, 5). This finding was surprising because PAR strain B241 had significantly higher expression of *rpoS* than B201 and B250, yet curli expression was higher in B201 and B250. This is in contrast to other studies showing that the Crl protein, required for curli production in most strains, up-regulates *rpoS* expression in response to temperature (Bougdour et al., 2004). RpoS then stimulates *mlrA* and thus *csgD* for curli production (Robbe-Saule et al., 2006; Ogasawara et al., 2009). Some strains of *E. coli* O157:H7 have been shown to differ in *rpoS* expression, with phage insertions in *mlrA* limiting curli production (Uhlich et al., 2013). Similar to our results, other research has shown that a sRNA, GcvB, helps activate RpoS as well as repress CsgD. Although we did not see statistical significance in *gcvB* expression between strains, we hypothesize that small change in sRNAs may cause large changes in target gene expression. This hypothesis is supported in both the RT-qPCR and global transcriptomics data via RNA-Seq, where the genes targeted by *gcvB, csgD*, and *hdeAB*, are affected according to previously reported gene regulation (Jin et al., 2009). These data support the regulatory pattern (i.e. lack of curli expression) we observed for PAR strain B241. While some PAR strains (B204, B244, B247, B264, and B269) had evidence of curli expression by the Congo red assay, the level of expression was evidently insufficient to result in flocculation.

**Figure 3:**
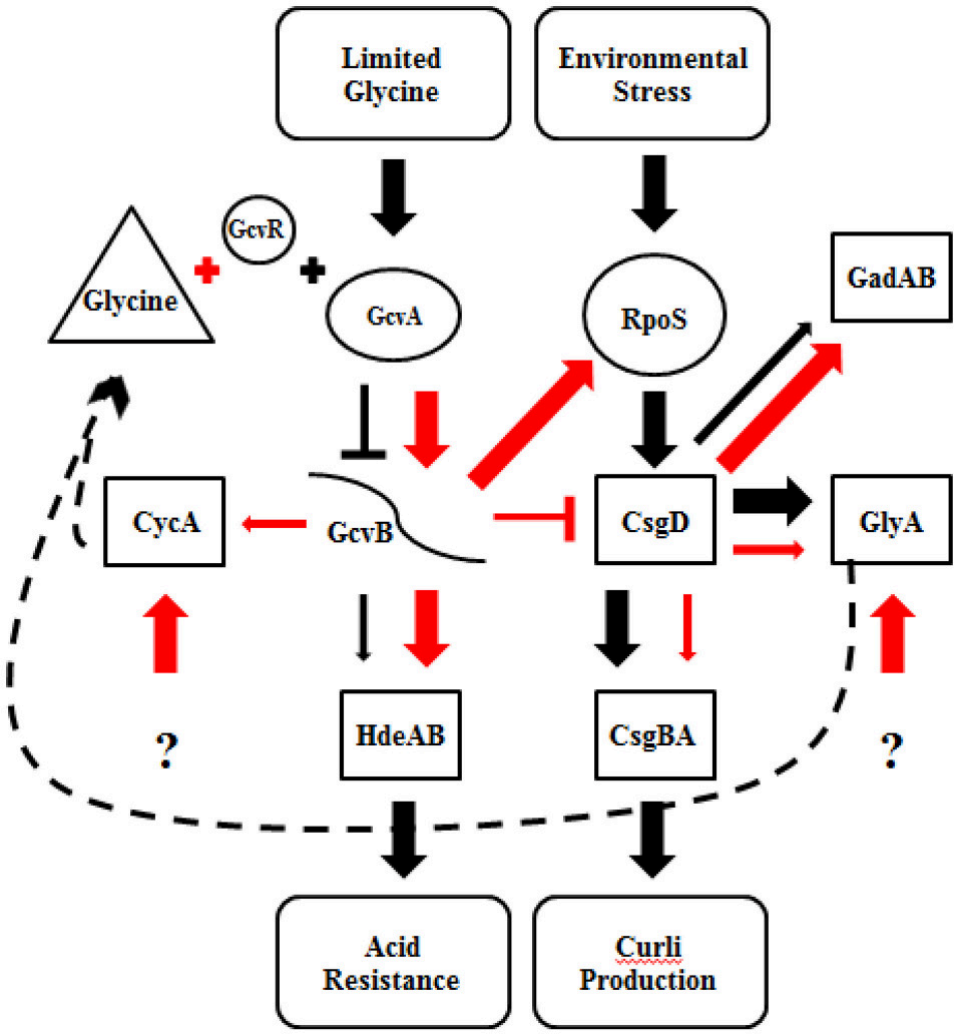
Regulatory model for curli expression and acid resistance phenotype of STEC strains B201 and B241. Proposed gene regulation of FAS (black) and PAR (red) STEC strains. The thickness of the arrows represents the amount of transcription, where thinner arrows indicate less transcription than thicker arrows. Barred lines represent transcription repression, the arrows increased transcription. The dashed line represents a proposed glycine feedback loop. Gene target categories are grouped by shape; regulatory genes (boxes), glycine cleavage system and RpoS (circles), sRNA (curved line), environmental conditions (oval), and glycine (triangle). The question mark indicates unknown effectors.

Curli production has also been linked to the presence of glycine (Urbanowski et al., 2000; Pulvermacher et al., 2008; Jin et al., 2009). When levels of glycine are high in the environment, GcvA activates GcvB, which represses CsgD and curli production. GcvB also directly represses *cycA*, which acts as a transporter of extracellular glycine into the cell, under nutrient rich conditions (Stauffer and Stauffer, 2012b). Acid induced chaperone genes, *hdeAB* and *rpoS*, are positively regulated by the *gcv* system (Stauffer and Stauffer, 2012a). When levels of glycine are low in the environment, GcvA and GcvR repress GcvB, which relieves repression of CsgD and curli production (Urbanowski et al., 2000; Jorgensen et al., 2012). CsgD in turn regulates GlyA, which converts serine to glycine. We found that supplementing M9GT with glycine suppressed the flocculation phenotype in B201 (Figure 1), indicating that curli expression was prevented. Unexpectedly, no differences in gene expression were seen between strains B201 and B201 supplemented with glycine even though the flocculation phenotype was relieved when grown in the presence of exogenous glycine. Fold-change observations are consistent with a model of gene regulation depicting the relationship between curli production in minimal medium and acid sensitivity (Figure 3). It is possible that a glycine-based feedback loop mediated by GcvB in B201 modulates curli expression, although other factors (indicated by question marks in Figure 3) may affect GcvB control of CycA and GlyA under nutrient limitation.

Previous findings for strains grown in complex medium show that GcvB represses GlyA due to CsgD repression (Stauffer and Stauffer, 2012b). The phenotypes observed may be due to selective pressure, where survival of acid sensitive strains under nutrient starvation may depend on biofilm formation (presaged by curli), whereas more acid resistant strains do not require this survival strategy. Previously, Oh et al. (2009) compared resistant and sensitive strains and found that strains of animal origin may (in general) be more acid resistant than food or human isolates, although acid resistance mechanisms were not addressed. Further research will focus on determining the effects of gene knockouts and complementation of target genes, *gcvB* and *hdeAB*, on FAS and PAR phenotypes, as well as the generation of metabolomic, proteomic, and additional transcriptomic data for these strains. These experiments may help to confirm our proposed model and possibly identify additional regulatory factors promoting curli formation in FAS and PAR STEC strains.

## Author contributions

KK, GK, and DO performed laboratory analysis of acid resistance of STEC strains. KK conducted the quantitative reverse transcriptase PCR. PF, GB, and GK conducted transcriptomic analysis and KK and GB contributed to statistical analysis of the data. FB, AG, PF, and DO contributed to the original conception and design of the work. All authors contributed to the drafting the manuscript and have agreed to support submission for publication.

### Conflict of interest statement

The authors declare that the research was conducted in the absence of any commercial or financial relationships that could be construed as a potential conflict of interest.
