# Supplementary material for: Escherichia coli O157:H7 Acid Sensitivity Correlates with Flocculation Phenotype during Nutrient Limitation
Source: Front Microbiol. 2017 Jul 26;8:1404. doi: 10.3389/fmicb.2017.01404 (PMC5526969; doi:10.3389/fmicb.2017.01404)
Supplement: Supplementary file 1 [file DataSheet1.docx]

***Escherichia coli* O157:H7 Acid Sensitivity Correlates with Flocculation Phenotype during Nutrient Limitation**

Running Title: acid sensitivity distinguished by curli

Journal Section: Food Microbiology

***Kathryn L. Kay^1,2^, Frederick Breidt^*1,2^, Pina M. Fratamico^3^, Gian Marco Baranzoni^3^, Gwang-Hee Kim^3,4^, Amy Grunden^1^, and Deog-Hwan Oh^4^***

*^1^Department of Plant and Microbial Biology, North Carolina State University, Raleigh, NC 27695; ^2^USDA-Agriculture Research Service, SAA Food Science Research Unit, 322 Schaub Hall NCSU, Raleigh, NC 27695; ^3^U.S. Department of Agriculture, Agricultural Research Service, Eastern Regional Research Center, 600 East Mermaid Lane, Wyndmoor, PA 19038, ^4^Department of Food Science and Biotechnology, College of Bioscience and Biotechnology, Kangwon National University, Hyoja 2-dong, Chuncheon 200-701, Republic of Korea*

Mention of a trademark or proprietary product does not constitute a guarantee or warranty of the product by the U. S. Department of Agriculture or North Carolina Agricultural Research Service, nor does it imply approval to the exclusion of other products that may be suitable. USDA is an equal opportunity provider and employer.

* Corresponding author Breidt; Phone: (919) 795-5657; email: fred.breidt@ars.usda.gov

**SUPPLEMENTAL TABLE 1.** Transcriptomic data for *E. coli* strain B241 in minimal medium.

| **GeneID^a^** | **201 Expression^b^** | **241 Expression^c^** | **LogFC (241 vs 201)^d^** | **Product** |
| --- | --- | --- | --- | --- |
| SEED:fig\|562.6424.peg.2611 | 0 | 265.8 | 9.056908618 | Chaperone HdeB |
| SEED:fig\|562.6424.peg.169 | 42.2 | 13024 | 8.252776204 | Glutamate decarboxylase (EC 4.1.1.15) |
| SEED:fig\|562.6424.peg.170 | 41.6 | 7468.9 | 7.471028316 | Probable glutamate/gamma-aminobutyrate antiporter |
| SEED:fig\|562.6424.peg.2617 | 39 | 6972.1 | 7.463700257 | Glutamate decarboxylase (EC 4.1.1.15) |
| SEED:fig\|562.6424.peg.2612 | 13.6 | 2011.5 | 7.156791332 | Chaperone HdeA |
| SEED:fig\|562.6424.peg.2580 | 6.5 | 381.8 | 5.771206468 | FIG00638244: hypothetical protein |
| SEED:fig\|562.6424.peg.1196 | 4.5 | 209.8 | 5.394376945 | hypothetical protein |
| SEED:fig\|562.6424.peg.2613 | 21.4 | 657.3 | 4.908646233 | Membrane transporter HdeD, H-NS repressed |
| SEED:fig\|562.6424.peg.2610 | 1.9 | 71.5 | 4.906890596 | Mg(2+) transport ATPase protein C |
| SEED:fig\|562.6424.peg.5403 | 0 | 12.6 | 4.711494907 | Hypothetical rem protein |
| SEED:fig\|562.6424.peg.4737 | 0.6 | 24.2 | 4.488935613 | Attachment invasion locus protein precursor |
| SEED:fig\|562.6424.peg.1044 | 1.3 | 38.7 | 4.444784843 | FIG00638505: hypothetical protein |
| SEED:fig\|562.6424.peg.1047 | 1.3 | 36.7 | 4.36923381 | FIG00638989: hypothetical protein |
| SEED:fig\|562.6424.peg.761 | 0 | 9.7 | 4.350497247 | Flagellar assembly protein FliH |
| SEED:fig\|562.6424.peg.287 | 1.3 | 33.8 | 4.252139765 | FIG00638731: hypothetical protein |
| SEED:fig\|562.6424.peg.1045 | 31.2 | 580 | 4.194741322 | Prophage Clp protease-like protein |
| SEED:fig\|562.6424.peg.3599 | 252.1 | 4421.2 | 4.129674601 | Chaperone protein DnaK |
| SEED:fig\|562.6424.peg.3107 | 38.3 | 549 | 3.823990924 | ATP-dependent protease HslV (EC 3.4.25.-) |
| SEED:fig\|562.6424.peg.218 | 0 | 5.8 | 3.655351829 | FIG00640134: hypothetical protein |
| SEED:fig\|562.6424.peg.1719 | 0 | 5.8 | 3.655351829 | Hydroxyaromatic non-oxidative decarboxylase protein C (EC 4.1.1.-) |
| SEED:fig\|562.6424.peg.2321 | 0 | 5.8 | 3.655351829 | probable membrane protein YPO3684 |
| SEED:fig\|562.6424.peg.5267 | 0 | 5.8 | 3.655351829 | Phage minor tail protein |
| SEED:fig\|562.6424.peg.774 | 100.1 | 1176.4 | 3.548289531 | putative cytoplasmic protein |
| SEED:fig\|562.6424.peg.1041 | 5.8 | 72.5 | 3.53447273 | Phage minor tail protein |
| SEED:fig\|562.6424.peg.3106 | 204.7 | 2343 | 3.513562159 | ATP-dependent hsl protease ATP-binding subunit HslU |
| SEED:fig\|562.6424.peg.654 | 3.2 | 39.6 | 3.438005061 | Nicotinamidase/isochorismatase family protein |
| SEED:fig\|562.6424.peg.1571 | 212.5 | 2301.5 | 3.433962498 | ClpB protein |
| SEED:fig\|562.6424.peg.843 | 0 | 4.8 | 3.40599236 | FIG00639155: hypothetical protein |
| SEED:fig\|562.6424.peg.3249 | 0 | 4.8 | 3.40599236 | FIG00638091: hypothetical protein |
| SEED:fig\|562.6424.peg.5265 | 0 | 4.8 | 3.40599236 | Phage minor tail protein |
|  |  |  |  |  |
| SEED:fig\|562.6424.peg.1046 | 46.8 | 481.4 | 3.348821712 | Phage portal protein |
| SEED:fig\|562.6424.peg.1370 | 0.6 | 10.6 | 3.334984248 | Aminopeptidase YpdF (MP-, MA-, MS-, AP-, NP- specific) |
| SEED:fig\|562.6424.peg.1042 | 7.8 | 83.1 | 3.332319701 | Minor tail protein Z |
| SEED:fig\|562.6424.peg.5280 | 9.1 | 95.7 | 3.324930583 | Protein yciF |
| SEED:fig\|562.6424.peg.2667 | 101.4 | 1014 | 3.315542908 | FIG00638451: hypothetical protein |
| SEED:fig\|562.6424.peg.2858 | 21.4 | 207.8 | 3.249660064 | 16 kDa heat shock protein A |
| SEED:fig\|562.6424.peg.1744 | 150.1 | 1429.6 | 3.247322356 | Phosphoadenylyl-sulfate reductase [thioredoxin] (EC 1.8.4.8) |
| SEED:fig\|562.6424.peg.3225 | 321 | 2957.8 | 3.20187772 | UPF0337 protein yjbJ |
| SEED:fig\|562.6424.peg.5281 | 24 | 224.3 | 3.197788381 | Conidiation-specific protein 10 |
| SEED:fig\|562.6424.peg.3600 | 65.6 | 592.5 | 3.165309928 | Chaperone protein DnaJ |
| SEED:fig\|562.6424.peg.501 | 0 | 3.9 | 3.137503524 | FIG00638395: hypothetical protein |
| SEED:fig\|562.6424.peg.1423 | 0 | 3.9 | 3.137503524 | Ethanolamine utilization protein EutG |
| SEED:fig\|562.6424.peg.1521 | 0 | 3.9 | 3.137503524 | 2,3-dihydroxy-2,3-dihydro-phenylpropionate dehydrogenase (EC 1.3.1.-) |
| SEED:fig\|562.6424.peg.1853 | 0 | 3.9 | 3.137503524 | Putative Type III secretion apparatus protein |
| SEED:fig\|562.6424.peg.2226 | 0 | 3.9 | 3.137503524 | Chaperone protein fimC precursor |
| SEED:fig\|562.6424.peg.2579 | 0 | 3.9 | 3.137503524 | FIG00638558: hypothetical protein |
| SEED:fig\|562.6424.peg.4263 | 0 | 3.9 | 3.137503524 | Apo-citrate lyase phosphoribosyl-dephospho-CoA transferase (EC 2.7.7.61) |
| SEED:fig\|562.6424.peg.4279 | 104.6 | 920.2 | 3.130968477 | Proposed lipoate regulatory protein YbeD |
| SEED:fig\|562.6424.peg.3429 | 121.5 | 1051.7 | 3.108455902 | Ribonucleotide reductase of class III (anaerobic), large subunit (EC 1.17.4.2) |
| SEED:fig\|562.6424.peg.1048 | 37 | 320.9 | 3.099407428 | Phage terminase, large subunit |
| SEED:fig\|562.6424.peg.683 | 117.6 | 990.8 | 3.069312766 | Trehalose-6-phosphate phosphatase (EC 3.1.3.12) |
| SEED:fig\|562.6424.peg.1636 | 9.1 | 79.3 | 3.055282436 | Carbon starvation induced protein CsiD |
| SEED:fig\|562.6424.peg.4128 | 139.1 | 1140.6 | 3.031054381 | Chaperone protein HtpG |
| SEED:fig\|562.6424.peg.5328 | 15.6 | 130.5 | 3.024434218 | Phosphoenolpyruvate-dihydroxyacetone phosphotransferase (EC 2.7.1.121), ADP-binding subunit DhaL |
| SEED:fig\|562.6424.peg.4399 | 111.8 | 904.7 | 3.010878657 | Probable secreted protein |
| SEED:fig\|562.6424.peg.3297 | 29.2 | 237.8 | 3.004244115 | PhnB protein; putative DNA binding 3-demethylubiquinone-9 3-methyltransferase domain protein |
| SEED:fig\|562.6424.peg.5327 | 26.6 | 216.5 | 3.001330286 | Phosphoenolpyruvate-dihydroxyacetone phosphotransferase (EC 2.7.1.121), subunit DhaM; DHA-specific IIA component / DHA-specific phosphocarrier protein HPr / DHA-specific EI component |
| SEED:fig\|562.6424.peg.649 | 1.3 | 13.5 | 2.959358016 | FIG00637868: hypothetical protein |

| **GeneID^a^** | **201 Expression^b^** | **241 Expression^c^** | **LogFC (241 vs 201)^d^** | **Product** |
| --- | --- | --- | --- | --- |
| SEED:fig\|562.6424.peg.2404 | 18.8 | 147.9 | 2.942818339 | tRNA 5-methylaminomethyl-2-thiouridine synthase TusB |
| SEED:fig\|562.6424.peg.624 | 22.7 | 174 | 2.911030326 | Ren protein |
| SEED:fig\|562.6424.peg.2344 | 0.6 | 7.7 | 2.898120386 | RND efflux system, membrane fusion protein CmeA |
| SEED:fig\|562.6424.peg.3296 | 0.6 | 7.7 | 2.898120386 | Phosphonate ABC transporter ATP-binding protein (TC 3.A.1.9.1) |
| SEED:fig\|562.6424.peg.4184 | 0.6 | 7.7 | 2.898120386 | FIG074102: hypothetical protein |
| SEED:fig\|562.6424.peg.2710 | 17.5 | 133.4 | 2.895087149 | FIG074102: hypothetical protein |
| SEED:fig\|562.6424.peg.680 | 1.3 | 12.6 | 2.863498 | Flagellar transcriptional activator FlhD |
| SEED:fig\|562.6424.peg.5635 | 1.3 | 12.6 | 2.863498 | Putative antirestriction protein |
| SEED:fig\|562.6424.peg.4772 | 148.2 | 1075.8 | 2.855603708 | DnaJ-class molecular chaperone CbpA |
| SEED:fig\|562.6424.peg.2301 | 5.2 | 40.6 | 2.85010457 | N-acetylmannosamine kinase (EC 2.7.1.60) |
| SEED:fig\|562.6424.peg.1745 | 243.7 | 1758.3 | 2.848456332 | Sulfite reductase [NADPH] hemoprotein beta-component (EC 1.8.1.2) |
| SEED:fig\|562.6424.peg.2347 | 28.6 | 205.9 | 2.826351913 | probable lipoprotein |
| SEED:fig\|562.6424.peg.2586 | 28.6 | 204.9 | 2.819345123 | Di/tripeptide permease DtpB |
| SEED:fig\|562.6424.peg.1043 | 5.2 | 39.6 | 2.814568412 | FIG00638599: hypothetical protein |
| SEED:fig\|562.6424.peg.2619 | 105.9 | 740.4 | 2.799780683 | Trehalase (EC 3.2.1.28); Periplasmic trehalase precursor (EC 3.2.1.28) |
| SEED:fig\|562.6424.peg.1253 | 0 | 2.9 | 2.765534746 | ElaD protein |
| SEED:fig\|562.6424.peg.3511 | 0 | 2.9 | 2.765534746 | RNA:NAD 2'-phosphotransferase |
| SEED:fig\|562.6424.peg.3927 | 0 | 2.9 | 2.765534746 | Putative inner membrane protein |
| SEED:fig\|562.6424.peg.3981 | 0 | 2.9 | 2.765534746 | Carbonic anhydrase (EC 4.2.1.1) |
| SEED:fig\|562.6424.peg.5329 | 29.2 | 201.1 | 2.762960803 | Phosphoenolpyruvate-dihydroxyacetone phosphotransferase (EC 2.7.1.121), dihydroxyacetone binding subunit DhaK |
| SEED:fig\|562.6424.peg.1776 | 1.3 | 11.6 | 2.748938236 | Glucarate dehydratase (EC 4.2.1.40) |
| SEED:fig\|562.6424.peg.4821 | 2.6 | 20.3 | 2.746243408 | Phage protein |
| SEED:fig\|562.6424.peg.188 | 0.6 | 6.8 | 2.73039294 | Formate dehydrogenase O beta subunit (EC 1.2.1.2) |
| SEED:fig\|562.6424.peg.3569 | 0.6 | 6.8 | 2.73039294 | FIG00638542: hypothetical protein |
| SEED:fig\|562.6424.peg.775 | 44.8 | 295.8 | 2.709475671 | Putative mannosyl-3-phosphoglycerate phosphatase (EC 3.1.3.70) |
| SEED:fig\|562.6424.peg.3376 | 75.4 | 481.4 | 2.66656201 | Acyl-CoA dehydrogenase (EC 1.3.8.7) |
| SEED:fig\|562.6424.peg.4745 | 16.9 | 106.3 | 2.617752436 | Uptake hydrogenase large subunit (EC 1.12.99.6) |
| SEED:fig\|562.6424.peg.1706 | 26.6 | 164.3 | 2.604351486 | Formate hydrogenlyase regulatory protein HycA |

| **GeneID^a^** | **201 Expression^b^** | **241 Expression^c^** | **LogFC (241 vs 201)^d^** | **Product** |
| --- | --- | --- | --- | --- |
| SEED:fig\|562.6424.peg.4771 | 72.8 | 428.2 | 2.548083314 | Chaperone-modulator protein CbpM |
| SEED:fig\|562.6424.peg.5415 | 1.9 | 13.5 | 2.544320516 | hypothetical protein |
| SEED:fig\|562.6424.peg.5157 | 3.9 | 25.1 | 2.540568381 | hypothetical protein |
| SEED:fig\|562.6424.peg.1422 | 0.6 | 5.8 | 2.517848305 | Ethanolamine permease |
| SEED:fig\|562.6424.peg.1454 | 0.6 | 5.8 | 2.517848305 | Formate hydrogenlyase subunit 4 |
| SEED:fig\|562.6424.peg.1930 | 0.6 | 5.8 | 2.517848305 | putative periplasmic protein kinase ArgK and related GTPases of G3E family |
| SEED:fig\|562.6424.peg.2581 | 0.6 | 5.8 | 2.517848305 | FIG00639317: hypothetical protein |
| SEED:fig\|562.6424.peg.3152 | 0.6 | 5.8 | 2.517848305 | FIG00639135: hypothetical protein |
| SEED:fig\|562.6424.peg.3469 | 0.6 | 5.8 | 2.517848305 | FIG00638866: hypothetical protein |
| SEED:fig\|562.6424.peg.3564 | 0.6 | 5.8 | 2.517848305 | hypothetical protein |
| SEED:fig\|562.6424.peg.3627 | 4.5 | 28 | 2.510961919 | Crotonobetainyl-CoA dehydrogenase (EC 1.3.99.-) |
| SEED:fig\|562.6424.peg.1730 | 13.6 | 79.3 | 2.500693584 | Putative cytochrome oxidase subunit |
| SEED:fig\|562.6424.peg.4011 | 146.2 | 821.6 | 2.486445023 | Alpha-ketoglutarate-dependent taurine dioxygenase (EC 1.14.11.17) |
| SEED:fig\|562.6424.peg.2622 | 40.3 | 227.2 | 2.480493234 | Inner membrane protein YhjD |

Global transcriptomics gene products of acid resistant (B241) *E. coli* O157:H7, denoted by coded GeneID^a^. Relative gene expression of B201^b^ and B241^c^ are listed with log fold change^d^ between the two strains. The 100 genes exhibiting the largest increase in expression for B201 (FAS) compared to B241 (PAR) are shown.

**SUPPLEMENTAL TABLE 2**. Transcriptomic data for *E. coli* strain B201 in minimal medium.

| **GeneID^a^** | **201 Expression^b^** | **241 Expression^c^** | **LogFC (241 vs 201)^d^** | **Product** |
| --- | --- | --- | --- | --- |
| SEED:fig\|562.6424.peg.4850 | 9165.9 | 1.9 | -11.89910512 | hypothetical protein |
| SEED:fig\|562.6424.peg.1078 | 757.7 | 0 | -10.56643465 | putative superinfection exclusion protein |
| SEED:fig\|562.6424.peg.1076 | 606.3 | 0 | -10.24507728 | putative membrane protein |
| SEED:fig\|562.6424.peg.1074 | 320.4 | 0 | -9.325979979 | Putative prophage repressor CI |
| SEED:fig\|562.6424.peg.5627 | 269.7 | 0 | -9.077883864 | Retron-type RNA-directed DNA polymerase (EC 2.7.7.49) |
| SEED:fig\|562.6424.peg.4835 | 261.2 | 0 | -9.031770114 | FIG00643399: hypothetical protein |
| SEED:fig\|562.6424.peg.1075 | 224.8 | 0 | -8.815703503 | FIG00643071: hypothetical protein |
| SEED:fig\|562.6424.peg.1077 | 223.5 | 0 | -8.807354922 | Phage antitermination protein N |
| SEED:fig\|562.6424.peg.5041 | 651.8 | 1 | -8.764429318 | Minor curlin subunit CsgB, nucleation component of curlin monomers |
| SEED:fig\|562.6424.peg.4779 | 199.5 | 0 | -8.64385619 | Phage integrase |
| SEED:fig\|562.6424.peg.1072 | 188.5 | 0 | -8.562242424 | Origin specific replication initiation factor |
| SEED:fig\|562.6424.peg.3909 | 1520 | 3.9 | -8.432826577 | CFA/I fimbrial auxiliary subunit |
| SEED:fig\|562.6424.peg.5042 | 1143.7 | 2.9 | -8.394588788 | Major curlin subunit precursor CsgA |
| SEED:fig\|562.6424.peg.4851 | 167 | 0 | -8.388017285 | Phage protein |
| SEED:fig\|562.6424.peg.4834 | 163.8 | 0 | -8.36018867 | Phage protein |
| SEED:fig\|562.6424.peg.2214 | 146.9 | 0 | -8.203592714 | hypothetical protein |
| SEED:fig\|562.6424.peg.1061 | 141 | 0 | -8.144658243 | Shiga toxin A-chain precursor (EC 3.2.2.22) |
| SEED:fig\|562.6424.peg.5575 | 139.7 | 0 | -8.131342539 | putative regulator; Regulation (Phage or Prophage Related) |
| SEED:fig\|562.6424.peg.4857 | 124.8 | 0 | -7.969242604 | Phage protein |
| SEED:fig\|562.6424.peg.5727 | 115.7 | 0 | -7.860466259 | Retron-type RNA-directed DNA polymerase (EC 2.7.7.49) |
| SEED:fig\|562.6424.peg.4856 | 768.8 | 2.9 | -7.821867752 | Phage protein |
| SEED:fig\|562.6424.peg.2160 | 108.5 | 0 | -7.768184325 | Protein Mom |
| SEED:fig\|562.6424.peg.1060 | 90.3 | 0 | -7.504620392 | Shiga toxin I subunit B precursor |
| SEED:fig\|562.6424.peg.4855 | 84.5 | 0 | -7.409390936 | FIG00639620: hypothetical protein |
| SEED:fig\|562.6424.peg.3910 | 2644.2 | 15.5 | -7.368888365 | CFA/I fimbrial major subunit |
| SEED:fig\|562.6424.peg.1073 | 76.7 | 0 | -7.270528942 | Phage repressor |

| **GeneID^a^** | **201 Expression^b^** | **241 Expression^c^** | **LogFC (241 vs 201)^d^** | **Product** |
| --- | --- | --- | --- | --- |
| SEED:fig\|562.6424.peg.4842 | 69.5 | 0 | -7.129283017 | Phage tail fiber protein |
| SEED:fig\|562.6424.peg.4845 | 60.4 | 0 | -6.928370323 | FIG00640812: hypothetical protein |
| SEED:fig\|562.6424.peg.5588 | 60.4 | 0 | -6.928370323 | FIG00640255: hypothetical protein |
| SEED:fig\|562.6424.peg.4801 | 57.2 | 0 | -6.850499414 | Phage repressor |
| SEED:fig\|562.6424.peg.4849 | 57.2 | 0 | -6.850499414 | Attachment invasion locus protein precursor |
| SEED:fig\|562.6424.peg.4830 | 53.3 | 0 | -6.749534268 | Lipoprotein Bor |
| SEED:fig\|562.6424.peg.4836 | 160.5 | 1 | -6.745954377 | conserved phage protein |
| SEED:fig\|562.6424.peg.3215 | 146.9 | 1 | -6.618630213 | Maltose/maltodextrin transport ATP-binding protein MalK (EC 3.6.3.19) |
| SEED:fig\|562.6424.peg.4846 | 47.4 | 0 | -6.581953751 | Phage tail fiber protein |
| SEED:fig\|562.6424.peg.4843 | 43.5 | 0 | -6.459431619 | FIG00641106: hypothetical protein |
| SEED:fig\|562.6424.peg.3911 | 718.1 | 7.7 | -6.453421215 | FIGfam014588: Predicted regulator of CFA/I fimbriae |
| SEED:fig\|562.6424.peg.1070 | 40.3 | 0 | -6.350497247 | Ren protein |
| SEED:fig\|562.6424.peg.4840 | 40.3 | 0 | -6.350497247 | FIG00642812: hypothetical protein |
| SEED:fig\|562.6424.peg.4852 | 39 | 0 | -6.303780748 | FIG00640276: hypothetical protein |
| SEED:fig\|562.6424.peg.1071 | 117 | 1 | -6.291554446 | Origin specific replication binding factor #replication protein P |
| SEED:fig\|562.6424.peg.2194 | 36.4 | 0 | -6.205548911 | putative transcription regulator |
| SEED:fig\|562.6424.peg.3908 | 3038.6 | 41.6 | -6.173680103 | CFA/I fimbrial subunit C usher protein |
| SEED:fig\|562.6424.peg.3907 | 1488.8 | 20.3 | -6.161907057 | CFA/I fimbrial minor adhesion |
| SEED:fig\|562.6424.peg.4837 | 33.8 | 0 | -6.100136671 | FIG00641463: hypothetical protein |
| SEED:fig\|562.6424.peg.2163 | 31.8 | 0 | -6.01346226 | Phage protein |
| SEED:fig\|562.6424.peg.2168 | 30.5 | 0 | -5.95419631 | Phage FluMu protein gp47 |
| SEED:fig\|562.6424.peg.2172 | 29.9 | 0 | -5.925999419 | Phage tail/DNA circulation protein |
| SEED:fig\|562.6424.peg.4832 | 29.9 | 0 | -5.925999419 | hypothetical protein |
| SEED:fig\|562.6424.peg.4839 | 29.9 | 0 | -5.925999419 | Phage protein |
| SEED:fig\|562.6424.peg.2171 | 29.2 | 0 | -5.892391026 | FIG003269: Prophage tail protein |
| SEED:fig\|562.6424.peg.2176 | 27.9 | 0 | -5.827819025 | Bacteriophage tail sheath protein |

| **GeneID^a^** | **201 Expression^b^** | **241 Expression^c^** | **LogFC (241 vs 201)^d^** | **Product** |
| --- | --- | --- | --- | --- |
| SEED:fig\|562.6424.peg.4838 | 27.9 | 0 | -5.827819025 | FIG00638618: hypothetical protein |
| SEED:fig\|562.6424.peg.283 | 83.8 | 1 | -5.812498225 | FIG00643946: hypothetical protein |
| SEED:fig\|562.6424.peg.2173 | 26 | 0 | -5.727920455 | Mu-like prophage FluMu protein gp42 |
| SEED:fig\|562.6424.peg.4804 | 25.3 | 0 | -5.689299161 | DNA helicase (EC 3.6.1.-), phage-associated |
| SEED:fig\|562.6424.peg.4860 | 25.3 | 0 | -5.689299161 | Phage EaA protein |
| SEED:fig\|562.6424.peg.5007 | 24.7 | 0 | -5.655351829 | Co-activator of prophage gene expression IbrB |
| SEED:fig\|562.6424.peg.4833 | 74.1 | 1 | -5.636141225 | Phage protein |
| SEED:fig\|562.6424.peg.4803 | 24 | 0 | -5.614709844 | Phage replication initiation protein #ACLAME10 |
| SEED:fig\|562.6424.peg.5220 | 22.7 | 0 | -5.5360529 | Phage terminase, large subunit |
| SEED:fig\|562.6424.peg.2174 | 21.4 | 0 | -5.452858965 | Mu-like prophage FluMu protein gp41 |
| SEED:fig\|562.6424.peg.4841 | 223.5 | 4.8 | -5.401362562 | Phage tail fiber protein |
| SEED:fig\|562.6424.peg.2161 | 20.1 | 0 | -5.364572432 | FIG00639839: hypothetical protein |
| SEED:fig\|562.6424.peg.4859 | 19.5 | 0 | -5.321928095 | Phage protein |
| SEED:fig\|562.6424.peg.4848 | 18.8 | 0 | -5.270528942 | FIG00641526: hypothetical protein |
| SEED:fig\|562.6424.peg.4783 | 18.2 | 0 | -5.224966365 | Phage EaA protein |
| SEED:fig\|562.6424.peg.1080 | 17.5 | 0 | -5.169925001 | Kil protein |
| SEED:fig\|562.6424.peg.2185 | 17.5 | 0 | -5.169925001 | Mu-like prophage FluMu protein gp29 |
| SEED:fig\|562.6424.peg.2195 | 17.5 | 0 | -5.169925001 | FIG00638221: hypothetical protein |
| SEED:fig\|562.6424.peg.4831 | 17.5 | 0 | -5.169925001 | Phage protein |
| SEED:fig\|562.6424.peg.4844 | 17.5 | 0 | -5.169925001 | hypothetical protein |
| SEED:fig\|562.6424.peg.5006 | 17.5 | 0 | -5.169925001 | Co-activator of prophage gene expression IbrA |
| SEED:fig\|562.6424.peg.2553 | 52 | 1 | -5.129283017 | FIG027190: Putative transmembrane protein |
| SEED:fig\|562.6424.peg.2166 | 16.9 | 0 | -5.121015401 | Prophage tail fiber protein |
| SEED:fig\|562.6424.peg.2212 | 16.9 | 0 | -5.121015401 | Phage transposase |
| SEED:fig\|562.6424.peg.5010 | 16.9 | 0 | -5.121015401 | Bundle-forming pilus protein BfpM |
| SEED:fig\|562.6424.peg.282 | 16.2 | 0 | -5.061776198 | FIG00638956: hypothetical protein |

| **GeneID^a^** | **201 Expression^b^** | **241 Expression^c^** | **LogFC (241 vs 201)^d^** | **Product** |
| --- | --- | --- | --- | --- |
| SEED:fig\|562.6424.peg.3906 | 525.1 | 15.5 | -5.037821465 | CFA/I fimbrial chaperone |
| SEED:fig\|562.6424.peg.157 | 15.6 | 0 | -5.008988783 | type 1 fimbrae adaptor subunit FimF |
| SEED:fig\|562.6424.peg.5026 | 15.6 | 0 | -5.008988783 | YeeU protein (antitoxin to YeeV) |
| SEED:fig\|562.6424.peg.5586 | 15.6 | 0 | -5.008988783 | DNA-cytosine methyltransferase (EC 2.1.1.37) |
| SEED:fig\|562.6424.peg.5016 | 72.8 | 1.9 | -4.932706887 | Antigen 43 precursor |
| SEED:fig\|562.6424.peg.4858 | 657.6 | 21.3 | -4.915906782 | Protein ygiW precursor |
| SEED:fig\|562.6424.peg.94 | 14.3 | 0 | -4.887525271 | Minor tail protein Z |
| SEED:fig\|562.6424.peg.2184 | 14.3 | 0 | -4.887525271 | Phage (Mu-like) virion morphogenesis protein |
| SEED:fig\|562.6424.peg.4853 | 14.3 | 0 | -4.887525271 | FIG00642416: hypothetical protein |
| SEED:fig\|562.6424.peg.5043 | 42.9 | 1 | -4.854660637 | Putative curli production protein CsgC |
| SEED:fig\|562.6424.peg.1228 | 13.6 | 0 | -4.817623258 | MFS Superfamily, Anion:Cation Symporter Family transporter |
| SEED:fig\|562.6424.peg.1886 | 13.6 | 0 | -4.817623258 | Accessory protein YqeC in selenium-dependent molybdenum hydroxylase maturation |
| SEED:fig\|562.6424.peg.2550 | 13.6 | 0 | -4.817623258 | (3R)-hydroxymyristoyl-[ACP] dehydratase (EC 4.2.1.-) |
| SEED:fig\|562.6424.peg.5037 | 13.6 | 0 | -4.817623258 | Curli production assembly/transport component CsgF |
| SEED:fig\|562.6424.peg.5451 | 174.8 | 5.8 | -4.798330357 | Uncharacterized fimbrial chaperone YehC precursor |
| SEED:fig\|562.6424.peg.2215 | 40.3 | 1 | -4.765534746 | PTS system, N-acetylgalactosamine- and galactosamine-specific IIA component (EC 2.7.1.69) |
| SEED:fig\|562.6424.peg.1014 | 13 | 0 | -4.754887502 | hypothetical protein |
| SEED:fig\|562.6424.peg.1792 | 13 | 0 | -4.754887502 | Fucose permease |
| SEED:fig\|562.6424.peg.2170 | 13 | 0 | -4.754887502 | Prophage baseplate assembly protein V |
| SEED:fig\|562.6424.peg.3885 | 13 | 0 | -4.754887502 | FIG054316: Phage polarity suppression protein |
| SEED:fig\|562.6424.peg.5012 | 13 | 0 | -4.754887502 | Mobile element protein |
| SEED:fig\|562.6424.peg.2341 | 12.3 | 0 | -4.678071905 | Adenine-specific methyltransferase (EC 2.1.1.72) |

Global transcriptomics gene products of acid sensitive (B201) *E. coli* O157:H7, denoted by coded GeneID^a^. Relative gene expression of B201^b^ and B241^c^ are listed with log fold change^d^ between the two strains. The 100 genes exhibiting the largest decrease (as negative values) in expression for B201 (FAS) compared to B241 (PAR) are shown.
